# A Case of Bilateral Idiopathic Temporal Cerebrospinal Fluid Leakage With Multiple Fistulas in the Middle and Posterior Cranial Fossae Closed Using the Transmastoid Approach

**DOI:** 10.7759/cureus.70742

**Published:** 2024-10-02

**Authors:** Shinya Ohira, Manabu Komori

**Affiliations:** 1 Department of Otolaryngology, St. Marianna University School of Medicine, Kawasaki, JPN

**Keywords:** idiopathic, obesity, posterior cranial fossa, sleep apnea, transmastoid approach

## Abstract

Although idiopathic temporal cerebrospinal fluid (CSF) leaks are a relatively rare condition, its incidence has been increasing in the United States in recent years. Fistulas commonly occur in the middle cranial fossa (MCF), and the MCF approach is recommended for multiple or large fistulas. Here, we present a case of bilateral temporal CSF leaks and multiple fistulas. The patient had small fistulas in the middle and posterior cranial fossae, which were successfully treated using the transmastoid (TM) approach, and proper postoperative management of CSF pressure resulted in favorable outcomes. The TM approach is particularly advantageous for addressing fistulas located in the posterior cranial fossa. Furthermore, in cases with multiple fistulas, a high likelihood exists that underlying factors such as idiopathic CSF hypertension, obesity, and obstructive sleep apnea (OSA) may be contributing to the condition. Managing these underlying factors effectively can lead to favorable outcomes. Despite the lower prevalence of idiopathic temporal CSF leak cases in Japan compared to the United States, the incidence of this condition is expected to rise globally owing to the increasing rates of obesity and OSA.

## Introduction

Idiopathic temporal cerebrospinal fluid (CSF) leaks are associated with risk factors such as obesity [1] and obstructive sleep apnea (OSA) [2], which are very common diseases in many countries. Although idiopathic CSF leaks are rare in Japan [3], the incidence has been increasing in the United States in recent years. Fistulas commonly occur in the middle cranial fossa (MCF), and the MCF approach is recommended for multiple or large fistulas [4]. The present case report is of a patient with bilateral temporal CSF leaks with multiple fistulas in the MCF and the posterior cranial fossa (PCF). Bilateral CSF leaks are a quite rare condition, and the fistulas were repaired using the transmastoid (TM) approach alone, resulting in a favorable outcome. There are several methods for closing CSF leaks, but to our knowledge, this is the first reported case of bilateral temporal CSF leakage closed using the TM approach only.

## Case presentation

**Figure 1:**
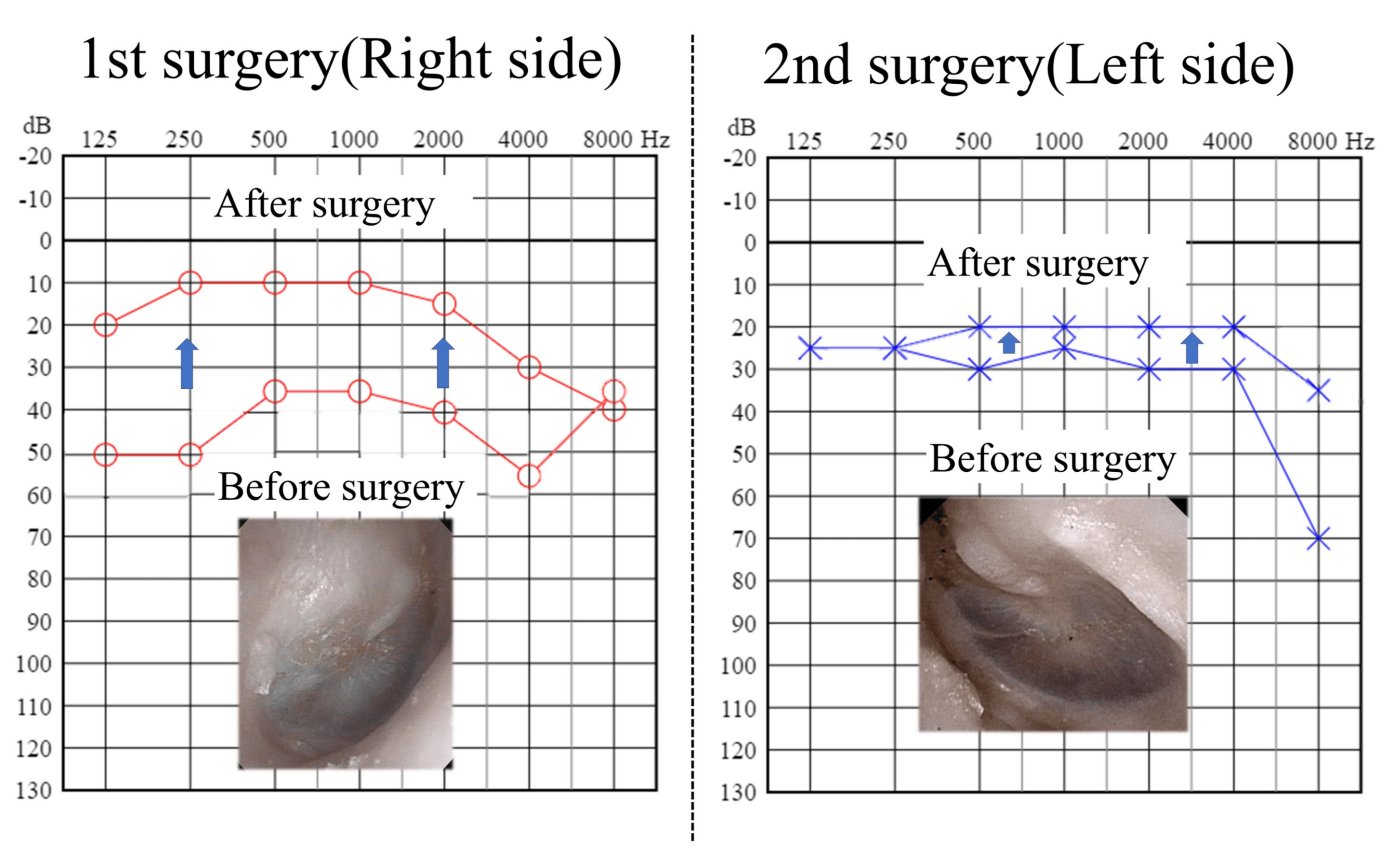
Findings, for the tympanic membrane, at the initial examination and change in pure tone audiometry (PTA) thresholds The tympanic membrane exhibited a pulsatile clear effusion; PTA improved after surgery in both the sides.

A 45-year-old male with a complaint of ear obstruction consulted his doctor and underwent tympanostomy for suspected right otitis media with effusion. Pulsatile serous otorrhea was observed through the incision, leading to a referral to our hospital with a suspected CSF leak. The patient had no history of head trauma, otologic surgery, or meningitis. He had started to gain weight around the age of 30 due to binge eating and drinking and lack of exercise. He had no habit of smoking. Upon initial examination, his height was 169 cm, weight 90 kg, and body mass index (BMI) 31.5 kg/m^2^. The right tympanic membrane exhibited a pulsatile clear effusion (Figure 1); no CSF was excreted from the pharyngeal orifice of the eustachian tube.

**Figure 2:**
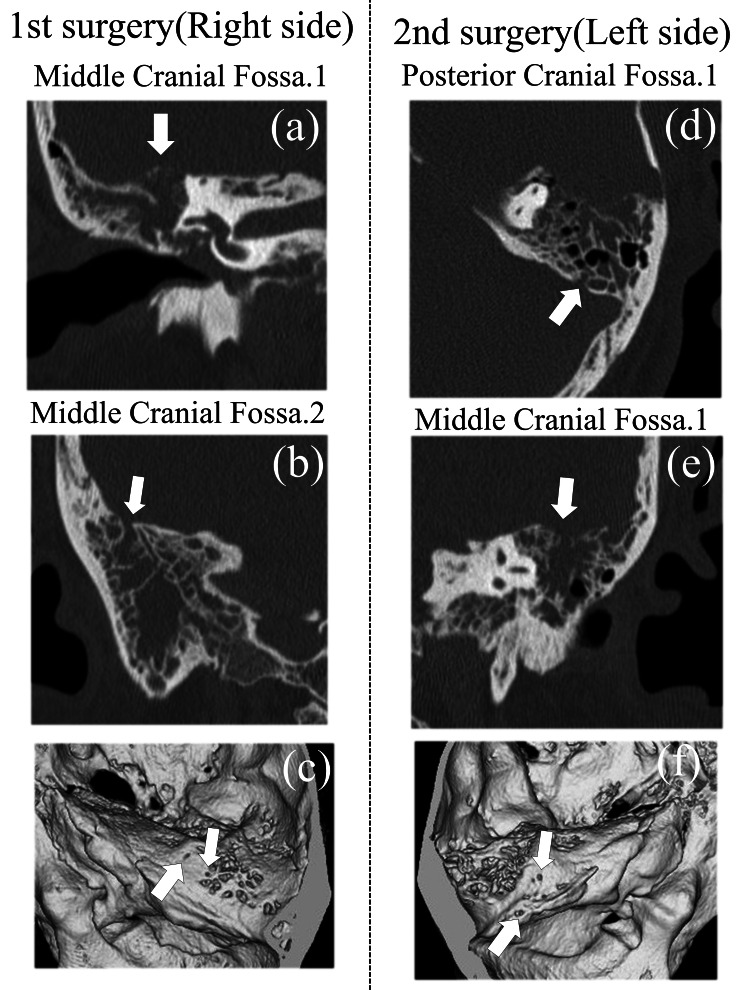
Preoperative computed tomography (CT) findings (a) A 2-mm bone defect in the middle cranial fossa at the level of the superior semicircular canal, (b) a 2-mm bone defect in the middle cranial fossa, slightly lateral to the first defect, (c) three-dimensional CT showing small bony defects in the right middle cranial fossa, (d) a 2-mm bone defect in the posterior cranial fossa, (e) a 2-mm bone defect in the middle cranial fossa, (f) three-dimensional CT showing small bony defects in the left posterior and middle cranial fossae.

Pure tone audiometry (PTA) showed right-sided conductive hearing loss with a threshold of 36.3 dB (using the quartering method) (Figure 1). The tympanogram was type B. Computed tomography (CT) scans revealed shadowing in the right tympanic cavity extending to the mastoid, along with a 2-mm bone defect in the MCF (Figures 2a, 2c).

**Figure 3:**
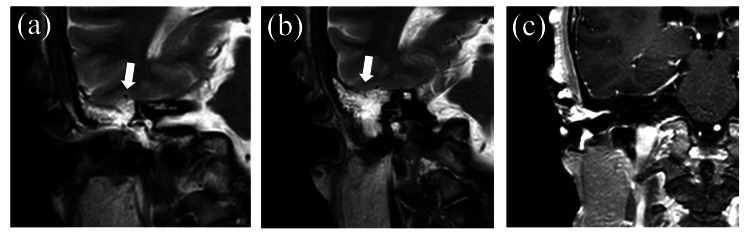
Preoperative magnetic resonance imaging (MRI) findings before the initial surgery (a) T2-weighted image showing hyperintensity in the region of computed tomography (CT) shading corresponding to the first fistula, (b) T2-weighted image showing hyperintensity in the region of CT shading corresponding to the second fistula, (c) no evidence of meningoencephalocele or arachnoid granulations on gadolinium-enhanced MRI.

Magnetic resonance imaging (MRI) showed a hyperintense area on T2-weighted images in the region of CT shading (Figures 3a, 3b), with no evidence of meningoencephalocele or arachnoid granulations (Figure 3c).

**Figure 4:**
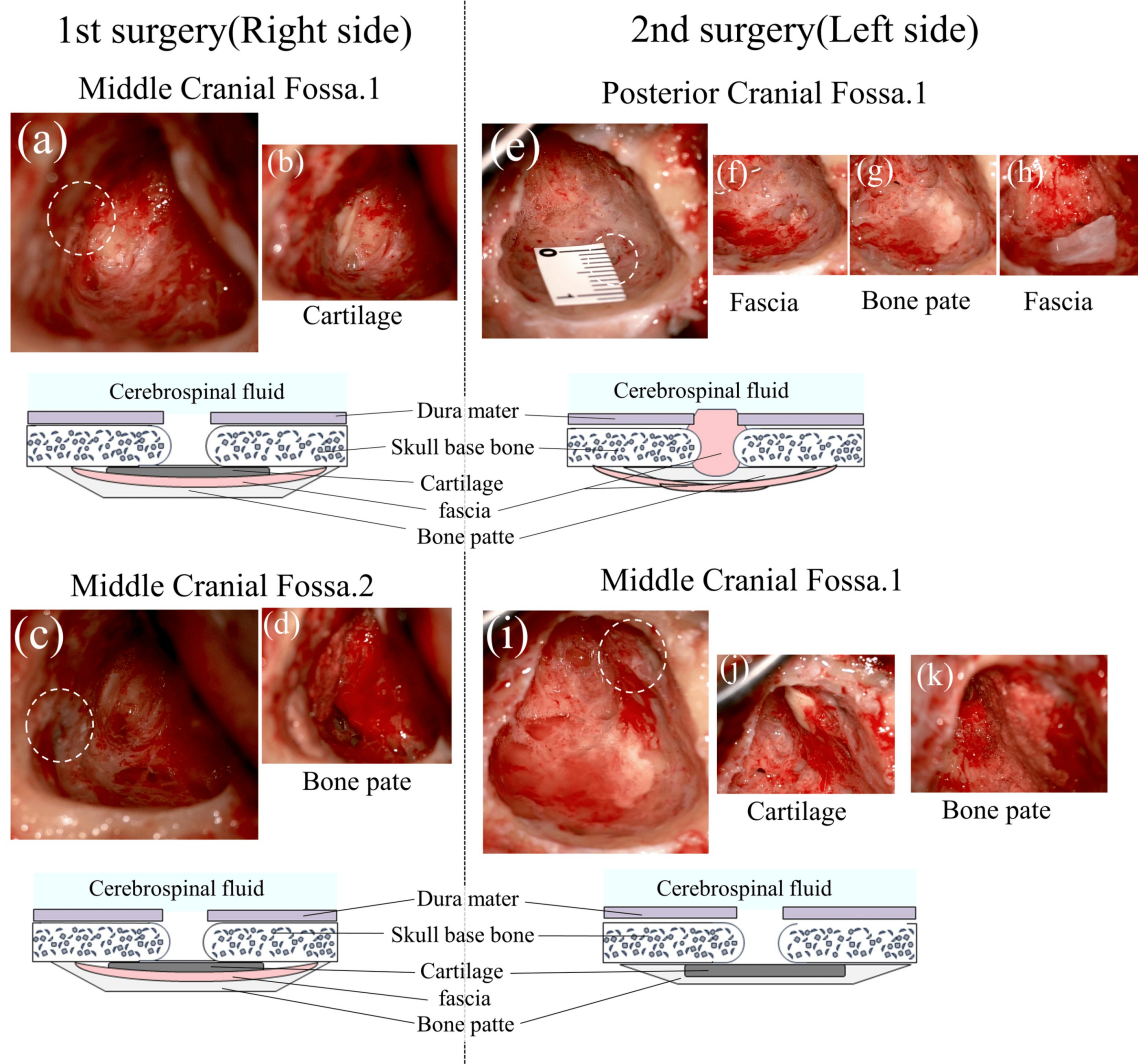
Surgical findings (a-d, right side; e-k, left side) (a) An 8 × 5 mm bony defect and cerebrospinal fluid (CSF) leak in the middle cranial fossa at the level of the superior semicircular canal. (b) The first layer of multilayer closure with thinly sliced cartilage for the first fistula. (c) A 3 × 3 mm bone defect and CSF leak, slightly lateral to the first defect. (d) The third layer of multilayer closure with bone pate for the second fistula. (e) A 2 × 2 mm bone defect in the posterior cranial fossa and CSF leak from the defect. (f) The first layer of multilayer closure with fascia inserted in the form of a “bath plug” for the first fistula. (g) The second layer of multilayer closure with bone pate for the first fistula. (h) The third layer of multilayer closure with fascia for the first fistula. (i) The 2 × 2 mm bone defect in the posterior cranial fossa and CSF leak from the defect. (j) The second layer of multilayer closure with thinly sliced cartilage for the second fistula. (k) The third layer of multilayer closure with bone pate for the second fistula.

Based on these findings, CSF leakage from a small fistula in the MCF was suspected, and the fistula was closed using a TM approach. At the surgery of the right side, mastoidectomy revealed an 8 × 5 mm bony defect in the MCF at the level of the superior semicircular canal (Figure 4a). A CSF leak was confirmed at this site and was repaired with multilayered closure using fascia, thinly sliced cartilage, and bone putty (Figure 4b). After closing the defect, a clear CSF leak was observed, prompting repositioning of the patient into a head-down position. Another CSF leak, located slightly lateral to the initial defect, was identified at a 3 × 3 mm bone defect with a fistula (Figure 4c), which was also repaired (Figure 4d). The surgery concluded after confirming no further leakage using the Valsalva maneuver. Preoperative CT had shown a bone defect at this same site (Figure 2b).

After the surgery, ear obstruction disappeared and the right-sided hearing of the patient improved to 11.3 dB (Figure 1), with no recurrence of the CSF leak. However, mild left-sided hearing loss was observed three years and four months postoperatively.

At a return visit, his weight was 94 kg and BMI 32.9 kg/m^2^. The left tympanic membrane exhibited a pulsatile clear effusion (Figure 1). No CSF was excreted from the pharyngeal orifice of the eustachian tube. Audiometry showed left-sided conductive hearing loss with a threshold of 27.5 dB (using the quartering method) (Figure 1). The tympanogram was type B. CT scans revealed shadows extending from the tympanic cavity to the mastoid and bone defects in the upper tympanic cavity, involving both the middle and posterior cranial fossae (Figures 2d-2f). The defects were approximately 2 mm in diameter.

Based on the aforementioned history, the patient was diagnosed with a left-sided temporal CSF leak. As multiple fistulas were observed in the previous surgery, we anticipated multiple fistulas in this case as well. However, as the fistulas were small and the previous surgery had been successful, we decided to proceed with a TM approach, suspecting a fistula in the PCF.

On surgery of the left side, mastoidectomy revealed a 2 × 2 mm bone defect in the middle and posterior cranial fossae (Figures 4e, 4i), with CSF leakage observed. The defects were sealed using fascia in a "bath-plug" configuration (Figure 4f) and further closed with thin cartilage (Figure 4j), bone putty (Figures 4g, 4k), and fascia (Figure 4h). After the surgery, although PTA improved to 20.0 dB (Figure 1), elevated CSF pressure (29 mmHg) was identified, consistent with idiopathic CSF hypertension. CSF pressure was managed medically, and the patient was advised to lose weight in view of his high BMI. According to his family, he was suffering from sleep apnea for the past four years; polysomnography (PSG) was performed that showed an apnea-hypopnea index of 85. He was diagnosed with severe OSA, and continuous positive airway pressure (CPAP) therapy was administered. The patient has been doing well, with no recurrence of symptoms in four years since his first visit.

## Discussion

Temporal CSF leaks are relatively rare and are most commonly caused by trauma, otitis media, or surgery [3]. However, cases of idiopathic temporal CSF leaks have been increasing in recent years. The average age of onset is 55-60 years, with no significant difference between sexes [5].

Two main mechanisms have been proposed for idiopathic CSF leaks: arachnoid granulations and increased CSF pressure [6]. Arachnoid villi are responsible for absorbing CSF, but abnormal arachnoid granulations can impair this function, leading to CSF retention, increased CSF pressure, and thinning of the underlying bone. Factors such as age-related dural fragility are also associated with CSF leakage. Thinning is particularly likely in patients with well-developed mastoid cells [5]. In the present case, while arachnoid granulations were absent, the patient exhibited good bilateral mastoid cell development.

The presence of arachnoid granulations does not always lead to CSF leaks [6], and other factors must be considered. Bone defects at the skull base may result from excessive bone resorption at the time of pneumatization of the mastoid cells [5], with age-related dural weakening and prolonged cranial hypertension contributing to bone erosion and CSF leakage [7]. Although these mechanisms likely play a role in the pathogenesis of idiopathic CSF leakage, several recent reports have highlighted obesity and OSA as additional risk factors [8]. Obesity increases the intrathoracic pressure, reducing venous return from the brain and raising CSF pressure. The recent increase in cases of idiopathic CSF leakage in the United States has paralleled increasing obesity rates [1], with the incidence nearly doubling over the past decade [9]. Large studies have shown that patients with idiopathic CSF leakage often have a BMI of 35.0 kg/m^2^ or higher [1]. Similarly, apneic attacks in OSA also increase cerebral blood flow and intracranial pressure, making OSA a potential risk factor for idiopathic intracranial hypertension [2]. A systematic review by Bakhsheshian et al. found that 16.9% of patients with idiopathic CSF leakage had OSA and that patients with OSA were 4.7 times more likely to develop a CSF fistula [10]. Although temporal bone CSF leaks remain rare in Japan, their incidence is rising alongside increasing rates of obesity and OSA, highlighting the importance of awareness across various countries.

Symptoms of temporal CSF leaks include ear obstruction and pulsatile tinnitus. Idiopathic intracranial hypertension, a potential cause of temporal CSF leaks, may present with a range of symptoms such as headache, dizziness, nausea, and visual impairment [11]. However, these symptoms of intracranial hypertension may not always manifest during active spinal fluid leakage, as intracranial pressure is reduced. If left undetected, temporal CSF leaks can lead to meningitis, warranting close attention. Persistent and recurrent middle ear effusions are commonly observed, and CSF leakage is often suspected when clear otorrhea persists after tympanostomy or tube placement [12]. In patients with middle ear effusion following an atypical course, the possibility of CSF leakage must be considered.

The diagnosis of idiopathic CSF leakage is typically made using imaging techniques such as CT and MRI, as well as β2-transferrin testing. CT is useful for identifying the size and location of bony defects, whereas T2-weighted MRI can clearly depict any associated cerebral herniation [13]. β2-transferrin testing is highly sensitive and specific for detecting CSF leakage [12], but its necessity in diagnosing temporal CSF leaks remains controversial, as diagnosis can often be made based on clinical course and imaging findings. Reported positive test rates vary widely, ranging from 55% to 75%, likely owing to the slow and intermittent nature of the leakage [12]. Furthermore, β2-transferrin testing may not be essential for diagnosing temporal CSF leaks, as obtaining CSF through the tympanic membrane carries an increased risk of meningitis [3].

Surgical closure of the site of leakage and reinforcement of the skull base are essential for treating temporal CSF leaks. In cases of traumatic CSF leakage, surgery is typically recommended if no improvement occurs after one to two weeks of rest. However, in idiopathic CSF leaks, early surgical intervention should be considered. The three primary surgical approaches are the TM approach, MCF approach, and a combined approach utilizing both [3]. The TM approach, which is the most familiar approach to otolaryngologists, allows simultaneous access to the middle ear and dura mater of the middle and posterior cranial fossae. This approach also has the advantage of being minimally invasive, avoiding elevation of the MCF dura mater [12]. However, if the fistula is located anterior to the tympanic cavity, manipulation of the ossicular chain is required; this approach is generally recommended for small defects, typically less than 1 cm in size [14]. It is important to pay attention to the size of the repair tissue relative to the size of the fistula, to prevent recurrence of CSF leakage or meningitis.

In contrast, the MCF approach is primarily used in neurosurgery. This approach provides a broader field of view for repairing dural and bony defects, so the risk of recurrence is very low. This technique is particularly useful for addressing multiple fistulas or larger defects exceeding 2 cm in size, which may be missed with the TM approach. It also allows for the manipulation of canal defects anterior to the tympanic cavity without disturbing the ossicular chain, making this approach suitable for patients without hearing loss and with defects anterior to the tympanic cavity [4]. Despite its benefits and capacity for extensive procedures, the MCF approach does not allow for the manipulation of the PCF and carries risks of temporal lobe injury, seizures, and hematomas owing to brain compression [10].

Therefore, a combination of the TM and MCF approaches has been used in some facilities. The TM approach is used to locate the fistula, which is subsequently repaired using minimal craniotomy through the MCF approach. As this combined approach provides excellent exposure to the medial area, it is highly effective for treating multiple and large fistulas, resulting in a very high success rate. In principle, the choice of surgical approach should be based on the size and location of the fistula.

For fistula closure, a multilayered technique is recommended, using both rigid (e.g., cartilage and bone) and soft materials (e.g., fascia and fat) [15]. Three patterns of graft insertion sites exist: epidural, intradural, and a combination of both; however, no significant difference in outcomes has been observed. Epidural reconstruction is preferred as it reduces the risk of cerebral complications associated with intradural procedures. The bath-plug technique with soft tissue is safer for intradural insertion, and hard tissue should be inserted into the epidural space if there is sufficient space between the skull base and dura mater; the MCF approach is easier, when it comes to insertion into the dura mater, while the TM approach exposes less space of the dura mater from the skull base, and it is easier to fix the tissue.

Furthermore, the postoperative management of CSF pressure is critical. Once the leak is sealed, CSF pressure increases, and improper management can lead to early recurrence [8]. Consequently, performing a lumbar puncture is recommended at two to six weeks after surgery to assess CSF pressure [11]. Studies have reported a high recurrence rate when an increase of 20 cm H_2_O or more in CSF pressure is observed [16]. Management strategies for elevated CSF pressure include diuretic medication, spinal drainage, and addressing contributing factors such as obesity and OSA. In general, spinal drainage is recommended for cases with recurrence, a history of idiopathic CSF hypertonia, and high CSF pressure greater than 25 cm H_2_O. However, the effectiveness of spinal drainage for temporal CSF leaks remains controversial. Proper control and follow-up of CSF pressure are necessary to ensure optimal postoperative outcomes.

During the initial operation, a small fistula in the MCF on the unilateral side was suspected. Two fistulas were subsequently identified and repaired intraoperatively. The patient had a favorable postoperative course of two years before experiencing a recurrence on the contralateral side. Although multiple fistulas were again suspected, surgery was performed using the TM approach in view of the success of the previous operation and CT findings suggesting a fistula in the PCF. A fistula was subsequently confirmed and repaired at this site. In cases of small fistulas, reliable determination of the site of leakage using preoperative CT scans is challenging. The TM approach, which provides access to both the middle and posterior cranial fossae, is preferable when findings suggest involvement of the PCF. In addition, the patient exhibited elevated CSF pressure postoperatively, which was managed conservatively using weight loss and OSA control with CPAP therapy, leading to a favorable outcome.

Only a few reports of bilateral temporal CSF leakage are available; to the best of our knowledge, only six cases, including this case, have been reported so far (Table 1) [5,11,17-19]. Honda et al. reported successful management of bilateral CSF leakage with multiple fistulas and meningoencephaloceles using a combined approach for closure [5]. Furthermore, Zakaryan et al. reported a case of temporal CSF leakage from the PCF that was closed using a retrosigmoid (RS) approach [11]. Their team, with limited experience with the TM approach, concluded that the RS approach was less favorable owing to its invasiveness and lower certainty of closure. In idiopathic cases, the TM approach is often preferred in view of the possibility of finding a fistula in the PCF intraoperatively. Although the follow-up period in this case was longer than previous reports, the period from the second surgery was a little short, so careful follow-up is necessary.

**Table 1:** Bilateral temporal CSF leakage cases Y: year(s), M: months, MCF: middle cranial fossa, PCF: posterior cranial fossa, TM: transmastoid To date, only six cases have been reported. To our knowledge, this is the first report of multiple small fistulas in the middle and posterior cranial fossae with favorable outcomes using the transmastoid approach alone. *Period from initial surgery to second surgery.

No.	Author	Year	Age	Gender	Site		Single/Multiple		Size		Period*	Complication		Approach		Follow-up
					Right	Left	Right	Left	Right	Left		Right	Left	Right	Left	
1	Ferguson et al. [17]	1986	50	Female	MCF	MCF	Single	Multiple	＜1 cm	＜1 cm	2M	None	None	TM	TM	1Y2M
2	Lundy et al. [18]	1996	41	Female	Unclear	Unclear	Unclear	Unclear	Unclear	Unclear	7Y	Unclear	Unclear	Combined	Combined	Unclear
3	Lundy et al. [18]	1996	73	Male	Unclear	Unclear	Unclear	Unclear	Unclear	Unclear	1Y	Unclear	Unclear	MCF	Combined	Unclear
4	Merchant and McKenna [19]	2000	62	Male	MCF	MCF	Multiple	Multiple	＜1 cm	＜1 cm	2M	Meningocele	Meningocele	Combined	Combined	1Y2M
5	Honda et al. [5]	2004	49	Male	MCF	MCF	Single	Multiple	＞1 cm	＜1 cm	5M	Meningoencephalocele	None	Combined	Combined	3Y5M
6	Ohira and Komori (present case)	2024	45	Male	MCF	MCF+PCF	Multiple	Multiple	＜1 cm	＜1 cm	3Y4M	None	None	TM	TM	4Y

To our knowledge, this is the first reported case of bilateral temporal CSF leakage with multiple small fistulas in the middle and posterior cranial fossa with a favorable outcome using the TM approach alone. We emphasize the importance of selecting an appropriate surgical approach and managing CSF pressure postoperatively to achieve favorable outcomes with minimal invasiveness.

## Conclusions

Although the MCF approach is recommended for patients with multiple fistulas, the TM approach is particularly advantageous for addressing fistulas located in the PCF. Furthermore, in cases with multiple fistulas, a high likelihood exists that underlying factors such as idiopathic CSF hypertension, obesity, and OSA may be contributing to the condition. Managing these underlying factors effectively can lead to favorable outcomes. Despite the lower prevalence of idiopathic temporal CSF leak cases in Japan compared to the United States, the incidence of this condition is expected to rise globally owing to the increasing rates of obesity and OSA.
